# Glucagon-Like Peptide-1 Receptor Agonist Ameliorates 1-Methyl-4-Phenyl-1,2,3,6-Tetrahydropyridine (MPTP) Neurotoxicity Through Enhancing Mitophagy Flux and Reducing α-Synuclein and Oxidative Stress

**DOI:** 10.3389/fnmol.2021.697440

**Published:** 2021-07-07

**Authors:** Tsu-Kung Lin, Kai-Jung Lin, Hung-Yu Lin, Kai-Lieh Lin, Min-Yu Lan, Pei-Wen Wang, Tzu-Jou Wang, Feng-Sheng Wang, Po-Chin Tsai, Chia-Wei Liou, Jiin-Haur Chuang

**Affiliations:** ^1^Center for Mitochondrial Research and Medicine, Kaohsiung Chang Gung Memorial Hospital and Chang Gung University College of Medicine, Kaohsiung, Taiwan; ^2^Department of Neurology, Kaohsiung Chang Gung Memorial Hospital and Chang Gung University College of Medicine, Kaohsiung, Taiwan; ^3^Center of Parkinson’s Disease, Kaohsiung Chang Gung Memorial Hospital and Chang Gung University College of Medicine, Kaohsiung, Taiwan; ^4^Research Assistant Center, Show Chwan Memorial Hospital, Changhua, Taiwan; ^5^Department of Anesthesiology, Kaohsiung Chang Gung Memorial Hospital and Chang Gung University College of Medicine, Kaohsiung, Taiwan; ^6^Department of Metabolism, Kaohsiung Chang Gung Memorial Hospital and Chang Gung University College of Medicine, Kaohsiung, Taiwan; ^7^Department of Pediatric, Kaohsiung Chang Gung Memorial Hospital and Chang Gung University College of Medicine, Kaohsiung, Taiwan; ^8^Department of Medical Research, Kaohsiung Chang Gung Memorial Hospital, Kaohsiung, Taiwan; ^9^Department of Pediatric Surgery, Kaohsiung Chang Gung Memorial Hospital and Chang Gung University College of Medicine, Kaohsiung, Taiwan

**Keywords:** GLP-1, autophagy flux, Parkinson disease, mitochondrial morphology, α-synuclein, diabetes mellitus, mitochondrial dynamics, mega-mitochondria

## Abstract

Parkinson disease (PD) is the second most common neurodegenerative disease without known disease modification therapy to slow down disease progression. This disease has pathological features of Lewy bodies with α-synuclein aggregation being the major component and selective dopaminergic neuronal loss over the substantia nigra. Although the exact etiology is still unknown, mitochondrial dysfunction has been shown to be central in PD pathophysiology. Type 2 diabetes mellitus has recently been connected to PD, and anti-diabetic drugs, such as glucagon-like peptide-1 receptor agonists (GLP-1RAs), have been shown to possess neuroprotective effects in PD animal models. The GLP-1RA liraglutide is currently under a phase 2 clinical trial to measure its effect on motor and non-motor symptoms in PD patients. In this study, we used an acute 1-methyl-4-phenyl-1,2,3,6-tetrahydropyridine (MPTP) mouse model of PD to test the possible mechanism of the GLP-1RA liraglutide in the pathogenesis of PD. We show that the neurobehavioral and motor dysfunction caused by the mitochondrial complex I inhibitor, MPTP, can be partially reversed by liraglutide. The GLP-1RA can protect mice from apoptosis of substantia nigra neurons induced by MPTP. MPTP treatment led to imbalanced mitochondrial fusion and fission dynamics, altered mitochondrial morphology, impeded autophagy flux, increased α-synuclein accumulation, and elevated oxidative stress. Specifically, the normalizing of mitochondrial fusion-fission dynamic-related proteins and enhancement of autophagy flux after administration of liraglutide is associated with improving neuronal survival. This suggests that GLP-1RAs may provide potential beneficial effects for PD caused by mitochondrial dysfunction through improvement of mitochondrial morphology balance and enhancing damaged organelle degradation.

## Introduction

Parkinson disease (PD) is the second most prevalent neurodegenerative disease with two major neuropathological hallmarks: (i) neuronal loss in the substantia nigra leading to striatal dopamine deficiency; and (ii) the intraneuronal inclusion bodies known as Lewy bodies containing α-synuclein (α-syn) aggregates both in central and peripheral nerve systems (Poewe et al., 2017). Cardinal motor symptoms of PD include bradykinesia, tremor, rigidity, and postural instability. Non-motor PD symptoms include constipation and anosmia which may be associated with α-syn aggregation in the gut enteric nerves and the olfactory bulb (Chandra et al., 2017). Risk factors for PD include age, environmental factors, and genetic predisposition. However, the exact etiology of the disease has not yet been discovered. Until now, there is still no cure for the disease, and existing symptomatic treatments will not change the course of the disease, which makes it important to determine potential treatment targets (Charvin et al., 2018). To date, several lines of evidence implied that mitochondrial dysfunction is the major player in the pathogenesis of PD (Lin et al., 2019; Lin et al., 2020). As the main energy generator in cells, mitochondria are especially important in high energy demand cells such as neurons. The organelle also possesses key roles in cellular signaling, cell bioenergetics, and cell survival. The linkage between PD and mitochondria can be traced back to the 1980s when the PD research landscape discovered that recreational drug exposure of 1-methyl-4-phenyl-1,2,3,6-tetrahydropyridine (MPTP) caused parkinsonism through its mitochondrial toxic byproduct 1-methyl-4-phenylpyridinium (MPP^+^) (Langston, 2017). Since then, increasing studies have continued to demonstrate the roles of mitochondria in neurodegeneration. Mitochondrial complex I inhibitors, MPTP and rotenone, are commonly used in experimental PD models to produce similar pathology to the human condition symptomatically and biochemically. Our previous data revealed that targeting mitochondrial complex I provided neuroprotection in cellular and rodent PD models (Lin et al., 2014, 2018). Accordingly, these models are important to predict validity in identifying agents that may be clinically effective (Duty and Jenner, 2011). Furthermore, the primary role of mitochondria in PD pathogenesis was also supported genetically as mutations of mitochondria-related genes such as phosphatase and tensin homolog-induced kinase-1 (*PINK1), parkin*, and *DJ-1* were found in members of familial PD (Reynolds et al., 2019).

A mitochondrion consists of four components including the mitochondrial outer membrane (MOM), mitochondrial inner membrane (MIM), the intermembrane space in between these two membrane structures, and the matrix at the central part. Within the mitochondrial matrix, the organelle houses its own small set of double-stranded, circular mitochondrial DNA (mtDNA) that contains 37 genes encoding two rRNAs, 22 tRNAs, and 13 polypeptides. All 13 protein products are constituents of the enzyme complexes of the oxidative phosphorylation (OXPHOS) system located on the MIM where adenosine triphosphate (ATP) is produced (Taylor and Turnbull, 2005). Mitochondria are the major source of cellular energy ATP. The OXPHOS machinery includes four electron transport chain (ETC) enzyme complexes (complex I to complex IV) and the F1F0-ATP synthase (complex V). As electrons are transferred via the series of redox reactions through the ETC, energy is generated which drives protons through the complexes I, III, and IV from the mitochondrial matrix into the intermembrane space. This creates an electrochemical gradient, called the mitochondrial membrane potential, and drives protons from the intermembrane space through the F1F0-ATP synthase back into the matrix causing the production of ATP through phosphorylation (Zorova et al., 2018). During the electron transport, byproducts of reactive oxygen species (ROS) are generated causing oxidative damage to nearby proteins and mtDNA. Therefore, the mitochondria have also developed their own protective antioxidative mechanisms to prevent excessive oxidative stress.

Within a single mammalian cell, thousands of mitochondria continuously fuse and divide forming a dynamic network of discrete isolated organelles to a large continuous reticulum (Kornfeld et al., 2018). Enabling proper functioning of the mitochondria maintenance of the dynamic equilibrium is essential. In fusion, mitochondria allow complementation of proteins and genes, while in fission damaged and dysfunctional parts are segregated. The resulting debris is cleared off through autophagy, and when fragmented mitochondria are targeted for lysosomal autophagy it is known as mitophagy (Palikaras et al., 2018). The fusion machinery of the organelle includes the MIM optic atrophy 1 (OPA1) and MOM mitofusin 1 and 2 (MFN1 and 2) (Xie et al., 2018). The fission-related mitochondrial proteins include the core machinery dynamin-related protein 1 (Drp1) and the adapter mitochondrial fission protein 1 (Fis1) (Rappold et al., 2014). These morphological structures are fundamental to the functionality of the organelle and are highly responsive to the cellular state. Despite the increasing detail of experimental results describing mitochondrial networks, many aspects of the function of mitochondrial fission and fusion remain unclear (Hoitzing et al., 2015). An imbalance between mitochondrial fusion and fission leads to dysfunctional mitochondria and is observed in neurodegenerative disorders (Xie et al., 2018).

Mitochondria are also involved in the decision-making of cellular fate through the activation of mitochondrial-dependent apoptosis for the maintenance of organismal homeostasis under several apoptotic regulators such as anti-apoptotic and pro-apoptotic B cell lymphoma-2 (Bcl-2) family proteins on the MOM (Singh et al., 2019). Once the mitochondrial-dependent apoptosis is activated, a series of cellular events are induced including mitochondrial membrane potential depolarization, the release of pro-apoptotic proteins from mitochondria such as cytochrome c, auto-cleavage, and activation of apoptotic key executioners caspase 9, 3, and 7, and eventually lead to programmed cell death for cells under an extreme hazardous environment (Wang and Youle, 2009; Singh et al., 2019).

The linkage of type 2 diabetes mellitus (T2DM) and PD has been demonstrated by a large cohort study that showed that PD incidence increased in T2DM patients, suggesting possible shared genetic predisposition or pathogenic pathways for both diseases (De Pablo-Fernandez et al., 2018). Recently, there has been interest in the repurposing of clinically used drugs on off-labeled conditions and among them is the use of glucagon-like peptide-1 receptor agonists (GLP-1RAs), a medication for lowering blood sugar in T2DM, for PD (Aviles-Olmos et al., 2013b). GLP-1RAs are able to stimulate GLP-1 receptors which are naturally occurring peptides found throughout the brain, increase the incretin effect in patients with T2DM, stimulate the release of insulin, and has been demonstrated to provide neurotrophic and neuroprotective effects (Perry et al., 2002a,b). Supporting this, treatment with the GLP-1RA exenatide for 12 months in a single-blind open-label randomized controlled trial (RCT) in 45 patients demonstrated mean improvement in the MDS-Unified Parkinson’s Disease Rating Scale (MDS-UPDRS) by 2.7 points, compared with a mean decline of 2.2 points in control patients (Aviles-Olmos et al., 2013a). Another GLP-1RA, liraglutide, has been approved by the Food and Drug Administration (FDA) to treat adults with T2DM and to treat obesity. Currently, liraglutide is under a phase 2 investigational trial to test the efficacy and safety of this drug in the treatment of patients with idiopathic PD with impending trial results. The beneficial effect of GLP-1RAs on learning and memory in experimental animals has also been reported (During et al., 2003). The protective effect of GLP-1RAs in PD models was shown to involve the activation of GLP-1 receptors and the following: adenylyl cyclase activation, elevated intracellular cAMP, and activation of protein kinase A (PKA) and phosphoinositide 3-kinase (PI3K). Downstream pathways including (i) mitogen-associated protein kinase/extracellular signal-regulated kinase (MAPK/ERK); and (ii) PI3K/protein kinase B (AKT) pathways (Zhou et al., 2015; Athauda and Foltynie, 2016) were then activated and triggered following stress responses, autophagy activation, apoptosis regulation, and neuron survival (Santo and Paik, 2018). Additionally, GLP-1RAs enhanced the survival of dopaminergic neurons and improved motor function in an MPTP mice PD model through reducing neuro-inflammation (Kim et al., 2009; Li et al., 2009; Zhang et al., 2019). The GLP-1RAs semaglutide and liraglutide were both shown to rescue substantia nigra neurons from cell death by decreasing α-syn aggregation, a reduction in oxidative stress, and autophagy induction in chronic MPTP-induced PD mice (Zhang et al., 2019).

Comprehensive studies focusing on the protective effect of GLP-1RAs in association with mitochondria in an integrated view on substantia nigra neurons are scarce. In this study, we aim to simultaneously investigate the protective effect of the GLP-1RA liraglutide, an anti-diabetic drug approved by the U.S. Food and Drug Administration (FDA) in 2010, through analyzing the motor behavior of mice, dopaminergic neuron survival, apoptosis induction, mitochondrial dynamic balance, mitochondrial morphology, α-syn accumulation, and oxidative stress using an acute MPTP-induced PD mouse model.

## Materials and Methods

### Animals

Experiments were performed on specific pathogen-free male C57BL/6 mice (8 weeks old, weighing 22–25 g) obtained from the Experimental Animal Center of the National Science Council, Taiwan. All experimental protocols conducted on mice were performed in accordance with the standards established by the animal center of Kaohsiung Chang Gung Memorial Hospital. The animals were housed in stainless steel cages (single per cage) in an air-conditioned room (22 ± 2°C with a 12:12 h light: dark cycle [07:00–19:00]). Standard laboratory mice chow and tap water were available *ad libitum*. Mice were allowed 1 week of acclimatization. The experimental animal model of PD was induced by intraperitoneal (IP) administration of MPTP, a specific inhibitor of mitochondrial complex I.

### Drug Dose and Time Points

**FIGURE 1:**
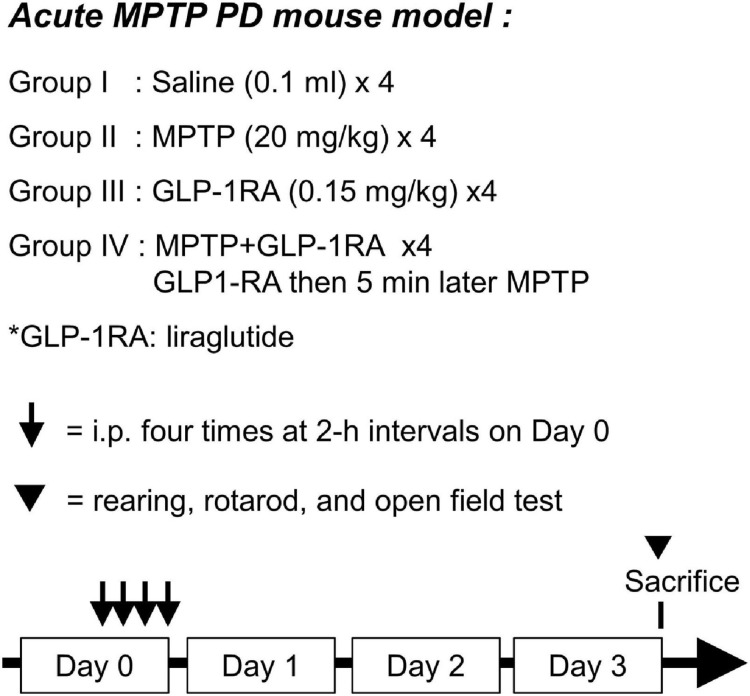
Schematic diagram of experimental design. Acute MPTP PD mouse model was used with 8-week-old C57BL/6 mice. The mice were allocated into four groups with the following treatments applied through intraperitoneal (ip) injection: group I, 0.1 ml of normal saline; group II, 20 mg/kg of MPTP; group III, 0.15 mg/kg of a GLP-1RA (liraglutide); group IV, co-treated with 0.15 mg/kg of the GLP-1RA then 5 min later 20 mg/kg of MPTP. This dose was repeated four times at 2-h intervals on Day 0. Three days after administration of the last dose, mice were subject to neurobehavior tests and then sacrificed. The substantia nigra was extracted for further experiments. GLP-1RA, glucagon-like peptide-1 receptor agonist.

Mice were divided into four groups at random: the control/MPTP/GLP-1RA/MPTP + GLP-1RA groups. Respective agents were applied to different groups for four doses via IP injection at 0, 2, 4, and 6 h as shown in Figure 1. For each dose, the control group was treated with 0.1 ml of normal saline, the MPTP group was treated with a 20 mg/kg dose of MPTP (1-Methyl-4-phenyl-1,2,3,6-tetrahydropyridine Hydrochloride, M325913; Toronto Research Chemicals, Canada Inc.), the GLP-1RA group was treated with 0.15 mg/kg of the GLP-1RA *liraglutide (Victoza)*, and the MPTP + GLP-1RA co-treatment group was treated with 20 mg/kg of MPTP and 0.15 mg/kg of GLP-1RA. Three days after the fourth dose was administered the mice were monitored for weight change, blood sugar levels, neurobehavior (RotaRod, rearing, and open field tests), and subject to tissue collection.

### RotaRod Test and Rearing Test

Motor coordination was assessed using a RotaRod (San Diego Instruments, San Diego, CA). Mice were placed on a horizontal bar [3.175 cm diameter (Dia.)] which rotates at a speed from 0 to 40 rpm in 5 min. Briefly, rats were trained to stay on the RotaRod apparatus during a 5 min habituation trial twice prior to the first testing day. The time to fall from the RotaRod was recorded. The average number of RotaRod tests over two trials was taken. A rearing test is used to evaluate the spontaneous motor initiation of mice. Animals were placed individually in a cylinder [8 cm (Dia.) × 12 cm (*H*)] and an observer counted the number of rearing events over 5 min. A rear was defined as lifting forepaws, typically to reach above the waistline and extending upward from hindlimbs, and was completed when forepaws returned to the floor of the cylinder. The average number of rears over two trials was taken.

### Open Field Test

We used the fear conditioning system (TSE, Bad Homburg) to test explorative locomotion in mice which were examined as the total distance traveled (m) and the number of rearing events during 15 min in a well-illuminated transparent acrylic cage (30 cm ^∗^ 30 cm) with a plain black floor, referred to as the open field. Open field tests were performed once for each mouse and data were analyzed with the multi conditioning system extended advanced 2.0.

### Immunohistochemistry Stain

The brain substantia nigra tissues were fixed with 10% formalin, dehydrated, and embedded in paraffin wax. Tissues were then sectioned to a thickness of 3 μm and placed on slides. The deparaffinization was completed by washing the slides in xylene three times, at 5 min each. The specimens were then rehydrated by washing slides in 100, 95, and 70% alcohol for 3 min each and gently rinsed using tap water. For blocking endogenous peroxidase activity, tissue sections were incubated with 3% H_2_O_2_ solution in methanol at room temperature for 10 min and then rinsed with phosphate buffer saline (PBS) twice, at 5 min each. Antigen retrieval was performed to unmask the antigenic epitope through a citrate buffer. Slides were arranged in a staining container, 300 ml of 10 mM citrate buffer, pH 6.0 was poured inside, and the container was incubated at 95–100°C for 30 min. The staining container was removed to room temperature and slides were cooled for 20 min. Slides were rinsed with PBS twice, at 5 min each. For immunohistochemistry and immunofluorescent staining, the tissue sections were incubated in a humidified chamber at room temperature for 1 h with 100 μl of appropriately diluted primary antibody [(anti-Tyrosine Hydroxylase antibody (ab137721), anti-active cleaved caspase 3 (ab32042), α-syn (ab209538; Abcam, Cambridge, England), Prohibitin Ab-1 (Clone II-14-10; Thermo Fisher Scientific, Cheshire, United Kingdom), 8-OHdG (sc-66036; Santa Cruz Biotechnology, Dallas, United States)]. Slides were rinsed with PBS for twice, at 5 min each. We then applied 100 μl of the appropriate secondary antibody ImmPRESS^®^-HRP universal polymer reagent (Horse Anti-Mouse/Rabbit igG, ZG0331, Vector Laboratories, Inc., Burlingame, CA 94010, United States) to the tissue sections on the slides and incubated them in a humidified chamber at room temperature for 1 h. Slides were rinsed with PBS twice, at 5 min each. 3,3′Diaminobenzidine (DAB) substrate solution 1:50 (ImmPACT^®^-DAB Substrate kit, Preoxidase, ZG1016, Vector Laboratories, Inc., Burlingame, CA 94010, United States) was applied and rinsed with PBS three times, at 2 min each. Slides were counterstained by immersing the slides in hematoxylin for 1 min and then rinsing them in running tap water for 10 min. Tissue slides were dehydrated by washing in alcohol four times (95, 95, 100, and 100%), at 5 min each. The tissue slides were cleared in xylene and a coverslip was applied using mounting solution. The mounted slides were stored at room temperature permanently. Observation of the antibody staining in the tissue sections was done under a fluorescent microscope (Olympus BX51). Quantification of the proteins was done by the staining intensity from three randomly chosen fields in each section and averaged using ImageJ software.

### TUNEL Stain

Substantia nigra cell apoptosis was detected via a terminal deoxynucleotidyl transferase 2′-Deoxyuridine, 5′-Triphosphate (dUTP) nick end labeling (TUNEL) assay using an *In Situ* Cell Death Detection kit, Fluorescein (Roche Diagnostics GmbH, Mannheim, Germany) according to the manufacturer’s protocol. The slides were mounted and the apoptotic cells were observed under a fluorescent microscope (Olympus BX51) and ≥ 3 fields of view at least were captured at 400× magnification. Quantification via staining intensity was done using ImageJ software.

### Western Blot Analysis

Tissue proteins were extracted from the substantia nigra samples with an extract buffer containing 0.5% Triton X-100 and protease-inhibitor cocktail (1:1,000, Sigma-Alsrich, St. Louis, MO). The tissues were homogenized in this buffer with the Fisher model 100 sonic dismembrator and put on ice for 1 h. The soluble extracts were separated by centrifugation at 13,898 × *g* for 5 min at 4°C. Equal amounts of protein samples (30 μg) were mixed with the loading buffer (60 mM Tris-HCl, 2% SDS, and 2% β-mercaptoethanol, pH 7.2), boiled for 4 min, resolved by SDS-polyacrylamide gels, and transferred to a nitrocellulose filter (Millipore, Bedford, MA) using a semidry blotting apparatus (Bio-Rad Laboratories, Hercules, CA). Membranes were blocked with 5% fat-free milk in Tris-buffered saline (TBS) and 0.05% Tween-20 (TBST) at room temperature for 1 h. The membranes were probed overnight at 4°C with primary antibodies (Anti-Tyrosine Hydroxylase antibody (ab75875), MFN-1 monoclonal antibody [11E91H12], mouse monoclonal 1:2,000 (ab126575), MFN-2 rabbit monoclonal 1:2,000 [NIAR164] (ab12477, Abcam, England), OPA1 (D6U6N) rabbit monoclonal antibody 1:2,000 (#80471), DRP1 (D6C7) rabbit monoclonal antibody 1:2,000 (#8570), Phospho-DRP1 (Ser616) Antibody 1:2,000 (#3455), LC3B rabbit polyclone antibody 1:2,000 (#2775), SQSTM1/p62 rabbit polyclone antibody 1: 10,000 (#5114, Cell Signaling Technology, Inc., United States); Fis1 (Cat No. GTX111010); GADPH rabbit polyclonal 1:10,000 (Cat No. GTX100118, Gene Tex Inc., United States). Detection of immunoreactive bands was performed with the appropriate secondary antibody goat anti-rabbit or goat anti-mouse conjugated with hydrogen peroxidase (Sigma-Aldrich, St. Louis, MO) and visualized by enhanced chemiluminescence according to the instructions of the manufacturer (Amersham Biosciences, Little Chalfont Buckinghamshire, England). Signal specificity was insured by omitting each primary antibody in a separate blot, and loading errors were corrected by measuring GAPDH immunoreactive bands in the same membrane. The density measurement of each band was performed with Scion image software (Scion Corporation, Frederick, MD). Background samples from an equivalent area near each lane were subtracted from each band to calculate mean band density.

### Statistical Analysis

All data were presented as the mean ± standard deviation and analyzed using Statistical Product and Service Solutions (SPSS) software version 17.0 (SPSS, Inc., Chicago, IL, United States). Differences among ≥ three groups were firstly evaluated using one-way analysis of variance (ANOVA), and if the differences were significant, multiple comparison analysis was performed. *P* < 0.05 was considered to indicate a statistically significant difference.

## Results

### Therapeutic Effect of the GLP-1RA on Behavior of MPTP-Induced PD Mice

**FIGURE 2:**
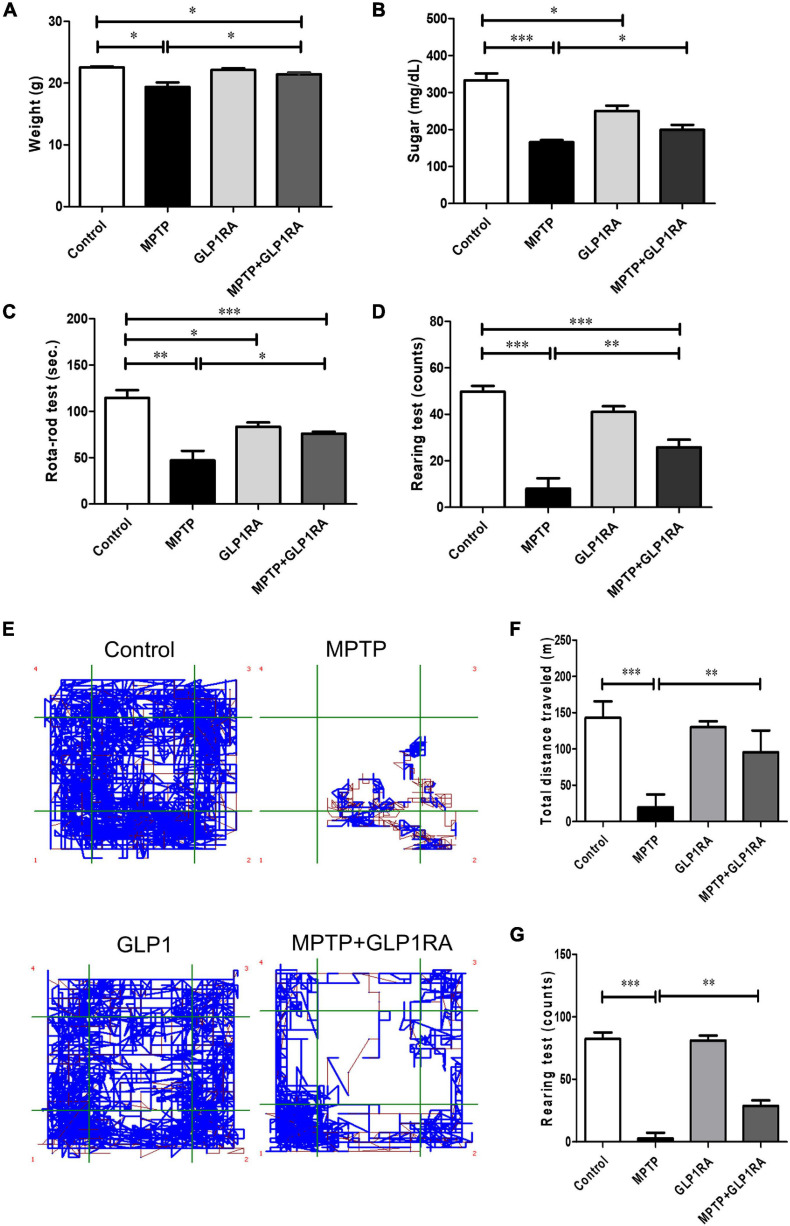
Therapeutic effect of the GLP-1RA (liraglutide) on behavior of MPTP-induced PD mice. Mice were allocated into four groups: control/MPTP/GLP-1RA/MPTP + GLP-1RA. **(A)** Mice weight: mice weight [gram (g)] was measured on the third day after the last dose of treatment. Control groups: *n* = 3 per group, treatment groups: *n* = 7 per group. **(B)** Blood sugar: mice blood sugar was checked on the third day after the last dose of treatment. Control groups: *n* = 3 per group, treatment groups: *n* = 7 per group. **(C)** The RotaRod test: Mice balance and coordination was measured through the RotaRod test where time to fall from a rotating rod at a speed accelerated from 0 to 40 rpm within 5 min was recorded. An average number of tests over two trials were taken. Control groups: *n* = 3 per group, treatment groups: *n* = 7 per group. **(D)** The rearing test: Spontaneous motor initiation and coordination alteration were observed via the rearing test. Mice were placed in an 8 cm (diameter) × 12 cm (height) cylinder and the number of rears were counted over 5 min. An average number of tests over two trials were taken. *n* = 3 per group for control groups and *n* = 7 per group for treatment groups. **(E–G)** The open field test: The explorative locomotion in mice was examined via the open field test. Mice were placed in a 30 cm * 30 cm acrylic cage and the total distance traveled [meters (m)] and number of rearing events over 15 min were measured to evaluate explorative locomotion. Control groups: *n* = 3 per group, treatment groups: *n* = 7 per group. The values represent mean ± SD (one-way ANOVA *P < 0.05, **p < 0.01, ***P < 0.001). GLP-1RA, glucagon-like peptide-1 receptor agonist.

The effect of a GLP-1RA on MPTP-induced PD mice on weight, blood sugar, and behavior was observed 3 days post four doses of normal saline/MPTP/GLP-1RA/MPTP + GLP-1RA (control/MPTP/GLP-1RA/MPTP + GLP-1RA groups) (Figure 2). MPTP caused significant weight reduction in experimental animals which could be recovered in the MPTP + GLP-1RA group. The GLP-1RA alone did not alter mice body weight (Figure 2A). Since this GLP-1RA is an anti-diabetic agent, we then checked the effect of this drug on blood sugar levels in experimental animals (Figure 2B). MPTP exposure significantly lowered blood sugar which may be directly due to neurotoxic effect and less feeding behavior caused by MPTP, and the reduced blood sugar was improved in the MPTP + GLP-1RA group. The anti-diabetic GLP-1RA only caused a mild though significant sugar lowering effect.

To determine the effect of the GLP-1RA on MPTP-induced PD mice neurobehavior, motor coordination and balance were quantified through the RotaRod performance test, rearing test, and the open field test on four groups after respective intervention on the 3rd day (Figures 2C–G). The balance time was reduced to 0.41-fold of the control in the MPTP group and there was a 1.6-fold increase in the MPTP + GLP-1RA group compared to the MPTP group (Figure 2C). Exposure of MPTP markedly decreased the balance time of the experimental mice on the RotaRod compared to control (47.0 ± 27.65 s vs. 114.7 ± 14.22 s). Treatment with the GLP-1RA on MPTP mice resulted in a significant improvement of performance on the RotaRod (75.9 ± 5.82 s). The GLP-1RA group had a mildly reduced balance time (83.3 ± 8.08 s) compared with the control group. Next, we manually counted rearing activity of mice, a behavior which is linked to striatal and dopaminergic activities (Schwarting et al., 1999; Figure 2D). MPTP greatly reduced mice rearing count (8.00 ± 11.89) in comparison to control (49.75 ± 4.99). The rearing count improved significantly in the MPTP + GLP-1RA group when compared to the MPTP group while the GLP-1RA alone did not cause significant disturbance to rearing counts (41.00 ± 4.36) compared to control. The 6.2-fold decrease in the MPTP group to control and 3.7-fold improvement in the MPTP + GLP-1RA group to MPTP group indicated that the GLP-1RA may provide protective effects. The open field test was then used to quantify locomotion of the animals by measuring the total distance traveled and rearing count automatically (Figures 2E–G). MPTP exposure induced a significant decrease in range of mobility and exploration into the open field map whilst the addition of the GLP-1RA to MPTP increased the range of mobility within the confined space. The total distance traveled after MPTP administration (19.75 ± 17.60 m) caused about a sevenfold decrease compared to control (143.00 ± 22.71 m) while the addition of the GLP-1RA to MPTP (95.58 ± 30.05 m) increased total distance traveled by 4.8-fold compared to the MPTP group. Together, these results revealed that a decrease of mice activity by MPTP can be partially alleviated by the GLP-1RA and that the mechanism for change in coordination and locomotion may be related to the dopaminergic pathway.

### The GLP-1RA Protects Dopaminergic Neurons From MPTP-Induced Cell Death

**FIGURE 3:**
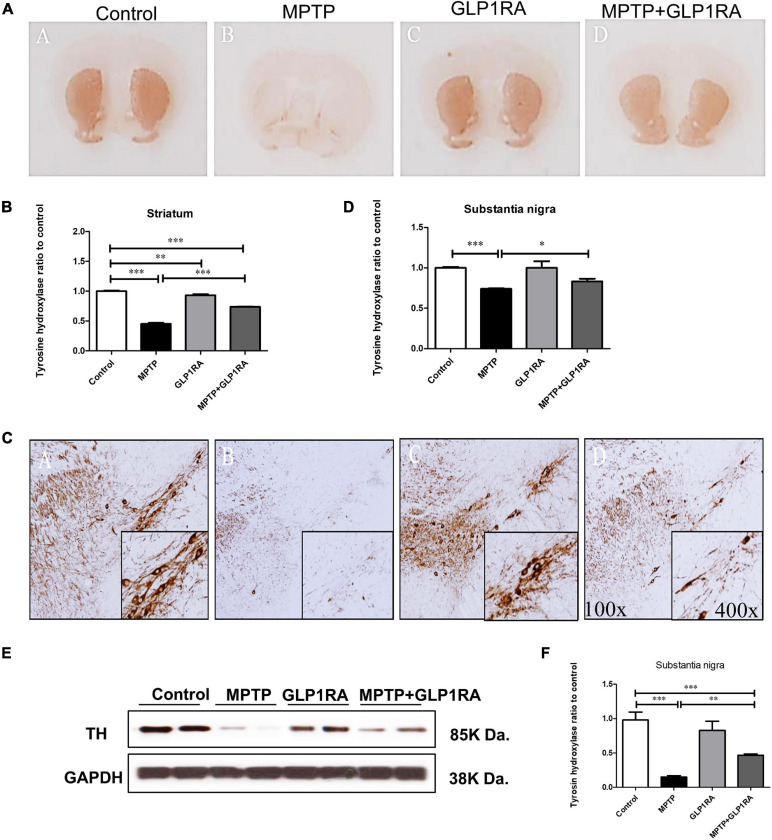
The GLP-1RA (liraglutide) protects dopaminergic neuron from MPTP-induced cell death. Immunohistochemistry (IHC) staining and immunoblotting of tyrosine hydroxylase (TH) in the striatum and substantia nigra were performed for the evaluation of viability of dopaminergic neurons. Mice were treated according to the four groups, control/MPTP/GLP-1RA/MPTP + GLP-1RA, and sacrificed on the 3rd day. **(A,B)** TH-IHC-stained striatum sections of the four groups (left to right: the control, MPTP, GLP-1RA, and MPTP + GLP-1RA groups) were viewed via a 4x light microscope and presented as TH staining intensity to percentage of the control, quantified using ImageJ software. *n* = 3 per group. **(C,D)** TH-IHC-stained substantia nigra sections of the four groups (left to right: the control, MPTP, GLP-1RA, and MPTP + GLP-1RA groups) were viewed via a 400x light microscope and presented as TH staining intensity to percentage of the control, quantified using ImageJ software. MPTP greatly reduced TH staining in comparison to control while the GLP-1RA partially reversed this. *n* = 3 per group. **(E,F)** Representative gels on immunoblotting of TH in the substantia nigra sections of the four groups. Protein expressions were normalized to GADPH as an internal standard, quantified using ImageJ software. Data are expressed as optical density to control. n = 4 per group. The values represent mean ± SD (one-way ANOVA, *P < 0.05, **p < 0.01, ***P < 0.001). GLP-1RA, glucagon-like peptide-1 receptor agonist.

After noting that GLP-1RA administration improved the neurobehavior of MPTP-exposed mice, we continued to examine if there were neuroprotective effects of the GLP-1RA on dopaminergic cell loss in the acute MPTP PD mouse. Tyrosine hydroxylase (TH) is a rate-limiting enzyme for dopamine synthesis; therefore, immunohistochemistry for TH can be used as an important marker of dopaminergic cell loss in the substantia nigra pars compacta (SNpc) (Xavier et al., 2005). Accordingly, our next experiment was to perform TH immunohistochemistry staining on the substantia nigra and striatum on the 3rd day over the four groups (Figure 3). Histopathologically, there was a 0.45-fold reduction of TH staining in the striatum of MPTP-exposed mice while the addition of the GLP-1RA to MPTP mice significantly reversed cell loss (Figures 3A,B). The immunohistochemistry for TH on the substantia nigra revealed a similar pattern and in relation to control, MPTP caused a 0.47-fold decrease in TH staining while the addition of the GLP-1RA to MPTP increased the TH staining by 0.64-fold in the substantia nigra compared to control (Figures 3C,D). To further confirm the neuroprotective effect of the GLP-1RA on MPTP-induced dopaminergic neuronal toxicity, Western blot was used to quantify the expression of TH in the substantia nigra (Figures 3E,F). MPTP exposure reduced TH expression levels by 0.15-fold in control which was partially alleviated in the MPTP + GLP-1RA mice (0.47 ± 0.02).

### The GLP-1RA Attenuated MPTP-Induced Apoptosis in the Substantia Nigra

**FIGURE 4:**
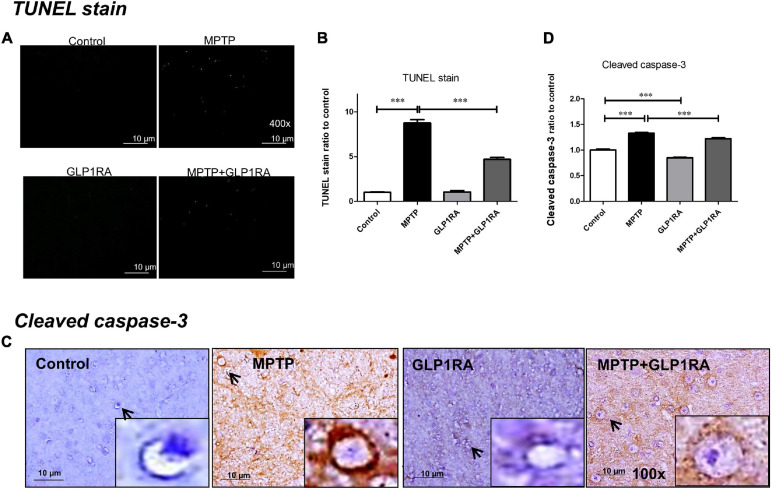
The GLP-1RA (liraglutide) attenuated MPTP-induced apoptosis in the substantia nigra. Mice were treated according to the four groups: control/MPTP/GLP-1RA/MPTP + GLP-1RA. **(A,B)** Terminal deoxynucleotidyl transferase-mediated dUTP-biotin nick end labeling (TUNEL) staining: TUNEL positive apoptotic cells of substantia nigra tissues were visualized under a fluorescent microscope 400x (green) and quantified via staining intensity to percentage of the control using ImageJ software. Scale bar = 10 μm. *n* = 3 per group. **(C,D)** Cleaved caspase-3 staining: IHC-staining of substantia nigra tissues were visualized under a light microscope 400x. Note that representative cells are denoted by arrows and caspase-3-positive neurons are identified by brown staining while the counterstain hematoxylin is shown in blue. Quantification through staining intensity was done using ImageJ software. Scale bar = 10 μm. *n* = 3 per group. The values represent mean ± SD (one-way ANOVA, *P < 0.05, **p < 0.01, ***P < 0.001). GLP-1RA, glucagon-like peptide-1 receptor agonist.

Showing that the GLP-1RA partially alleviated dopaminergic cell death in the MPTP PD model, we further examined the apoptosis of mice substantia nigra cells via TUNEL staining and of cleaved caspase-3 immunohistochemistry staining on mice substantia nigra tissue after four doses of normal saline/MPTP/GLP-1RA/MPTP + GLP-1RA on the 3rd day (Figure 4). Being a mitochondrial complex I specific inhibitor, MPTP exposure increased apoptosis by 8.77-fold (8.77 ± 0.37) and the addition of the GLP-1RA to MPTP mice reversed this trend significantly to 4.71-fold (4.71 ± 0. 20) in relation to control via TUNEL staining (Figures 4A,B). The GLP-1RA alone did not cause significant cellular apoptosis. Consistently, cleaved caspase-3 staining demonstrated that MPTP exposure increased neuronal apoptosis (1.33 ± 0. 02) and that the addition of the GLP-1RA to MPTP mice provided significant amelioration (1.22 ± 0. 02) in relation to control (Figures 4C,D). Overall, this suggests that the GLP1-RA has protective properties on substantia nigra neurons and that this protection may work through affecting mitochondrial-dependent apoptosis.

### The GLP-1RA Partially Normalizes Mitochondria Dynamic Imbalance and Enhances Impaired Autophagy Flux Induced by MPTP

**FIGURE 5:**
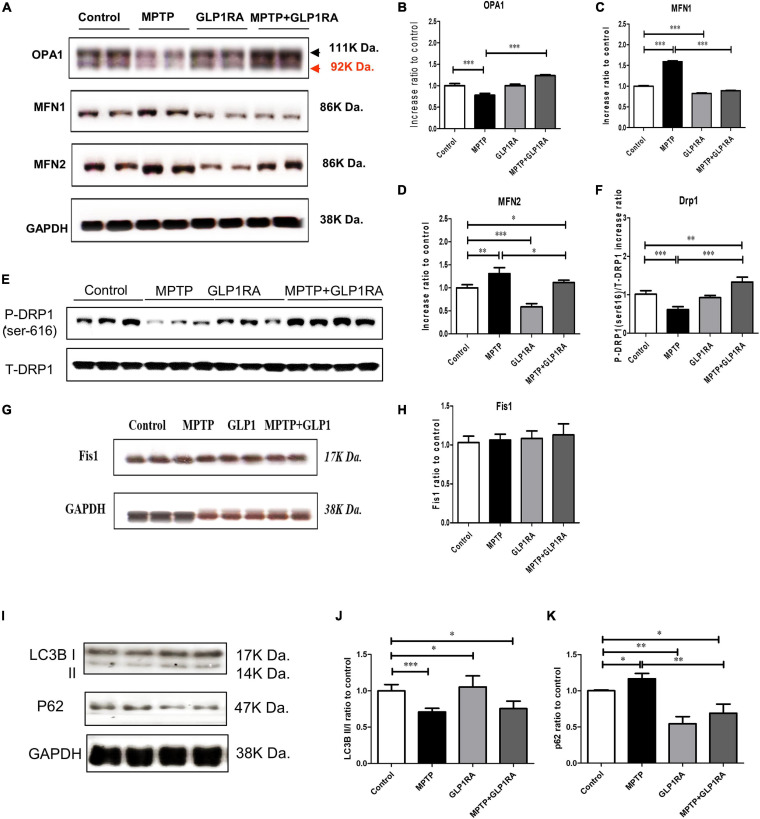
The GLP-1RA (liraglutide) partially normalizes mitochondria dynamic imbalance and restores MPTP-induced impaired autophagy flux. Mice were treated according to the four groups: control/MPTP/GLP-1RA/MPTP + GLP-1RA, and sacrificed on the 3rd day. Substantia nigra tissues were obtained. Representative Western blots and bar diagrams of the mitochondrial fusion regulatory proteins **(A,B)** OPA1, **(A,C)** MFN1, **(A,D)** MFN2, and fission regulatory proteins **(E,F)** pDRP1 and **(G,H)** Fis-1 of the four independent experiments (*n* = 4) are presented. The dysregulation of fusion and fission regulatory proteins levels due to MPTP exposure were rebalanced with GLP-1RA treatment. Representative Western blots and bar diagrams of **(I,J)** autophagy induction marker, LC3B, and **(I,K)** autophagy adapter, p62, of the three independent experiments (*n* = 3) are presented. The LC3B-II/LC3B-I ratio was reduced and p62 accumulated in the MPTP group while the GLP-1RA was able to decrease this p62 accumulation significantly suggesting that the GLP-1RA enhanced autophagy flux. Protein expressions were normalized to GADPH as an internal standard and quantified using Image J. The values represent mean ± SD (one-way ANOVA, *P < 0.05, **p < 0.01, ***P < 0.001). GLP-1RA, glucagon-like peptide-1 receptor agonist; OPA1, optic atrophy 1; MFN1 and 2, mitofusin 1 and 2; Drp1, dynamin-related protein 1; Fis1, mitochondrial fission protein 1; p62, p62/SQSTM1; LC3B, microtubule-associated proteins 1A/1B light chain 3B.

With the observation that the addition of a GLP-1RA to MPTP PD mice alleviates cellular apoptosis, we next studied mitochondrial morphology regulators and autophagy markers in the substantia nigra of the four groups, because mitochondria dynamic balance is crucial for the maintenance of mitochondrial homeostasis after stress induced by MPTP (Figure 5). Three days after respective treatment over the control/MPTP/GLP-1RA/MPTP + GLP-1RA groups, we measured the level of proteins involved in mitochondrial fusion: OPA1, MFN1, and MFN2, and mitochondrial fission proteins: DRP1 and Fis1 in tissues of the substantia nigra. The MIM fusion protein OPA1 was decreased by 0.78-fold (0.78 ± 0.04) in the MPTP group but increased by 1.24-fold (1.24 ± 0.02) after addition of the GLP-1RA to MPTP (Figures 5A,B). The MOM fusion proteins MFN1 and MFN2 were both elevated in the MPTP group (1.59 ± 0.04 and 1.31 ± 0.13, respectively) and the addition of the GLP-1RA to MPTP lowered both fusion protein levels (0.89 ± 0.01 and 1.12 ± 0.05, respectively) (Figures 5A,C,D). The reduced OPA1 and elevated MFN1 and MFN2 levels in MPTP-exposed mice may suggest a disturbed mitochondrial fusion after complex I inhibition. The administration of MPTP reduced the active form of the fission-related protein DRP1 p-ser616 (0.61 ± 0.08), and the addition of the GLP-1RA to MPTP significantly increased the active form of DRP p-ser616 (1.332 ± 0.13), although the total DRP1 remained unchanged (Figures 5E,F). The GLP-1RA alone did not produce a significant change in DRP p-ser616 levels. By MPTP or GLP-1RA administration, the other fission protein, Fis1, could recruit DRP1 to mitochondria, but there was no significant change in Fis1 levels (Figures 5G,H). These results indicate that MPTP may induce mitochondrial dynamics imbalance, and the GLP-1RA may partially restore its balance.

We continued to quantify autophagy activity by measurement of “autophagy flux” or the rate of autophagic degradation (Ueno and Komatsu, 2020) targeting two autophagy-related proteins, the microtubule-associated proteins 1A/1B light chain 3B (LC)3B and the p62/Sequestosome 1 (SQSTM1) (hereafter as p62). The lipidation of LC3-I (the cytoplasmic form of LC3) forms LC3-II, which is inserted into autophagosome membranes in the conversion process. Therefore, LC3B-II is an autophagosome membrane marker protein and has been established as a useful marker for autophagy induction (Liu et al., 2016). We checked the LC3-II/LC3-I ratio through immunoblotting and revealed that the LC3-II/LC3-I ratio (0.71 ± 0.05) of the MPTP group decreased, while the addition of the GLP-1RA did not alter this ratio significantly (0.76 ± 0.10) in the substantia nigra of mice (Figures 5I,J). The GLP-1RA alone increased the LC3-II/LC3-I ratio mildly to 1.05 ± 0.15 in comparison to control. Since the LC3-II/LC3-I ratio did not alter with the treatment of GLP-1RA, we checked the autophagy flux marker p62. With the induction of autophagy, LC3B-II can recognize adaptor proteins including p62, which bind selectively to substrates, including damaged mitochondrial structural proteins (Wang et al., 2016). During autophagy, p62-labeled damaged structures are degraded along with the recruited cargo. Therefore, the accumulation of p62 can be considered as a sign of reduced autophagy flux (Ueno and Komatsu, 2020). Exposure to MPTP increased p62 accumulation up to 1.17 ± 0.07-fold in comparison to control while the addition of the GLP-1RA to MPTP mice decreased p62 by 0.69 ± 0.14-fold, and GLP-1RA alone decreased p62 by 0.55 ± 0.10-fold (Figures 5I,K). This indicates that MPTP treatment impaired autophagy flux, while GLP-1RA administration restored the impaired autophagy flux. Taken together, the accumulation of p62 and the decrease of LC3-II/LC3-I ratio induced by MPTP exposure suggest that after the inhibition of mitochondrial complex I, the decrease in autophagy flux may lead to the loss of dopaminergic neurons, and the GLP-1RA will partially counteract this effect.

### Mega-Mitochondria Formation Noted With MPTP Treatment and Normalization of Mitochondrial Morphology With Administration of the GLP-1RA

**FIGURE 6:**
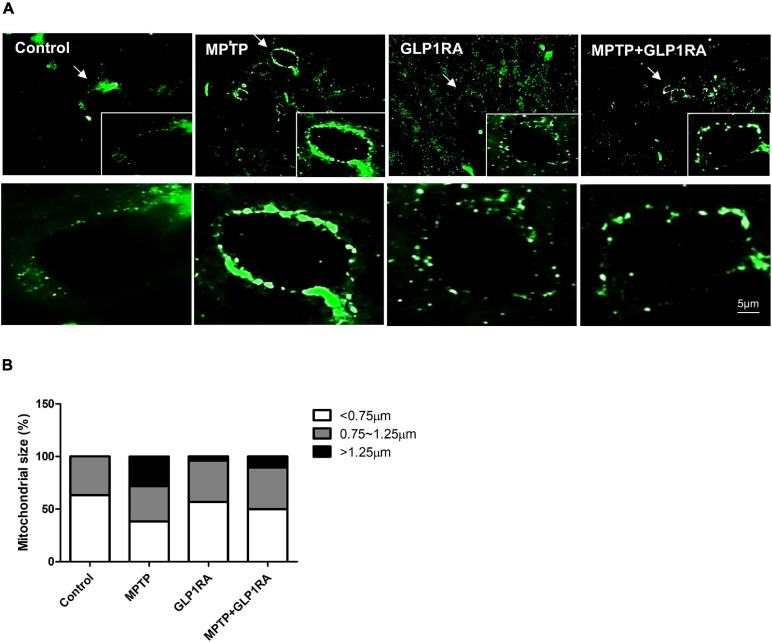
The effect of the GLP-1RA (liraglutide) and MPTP on the morphology of mitochondria in the substantia nigra. Mice were treated according to the four groups: control/MPTP/GLP-1RA/MPTP + GLP-1RA, and sacrificed on the 3rd day. Substantia nigra sections were obtained. **(A,B)** Representative images of substantia nigra sections immunofluorescence-stained with prohibitin, a mitochondrial scaffold protein (green). Note that representative cells are denoted by arrows. Image was visualized under a 1000x oil fluorescent microscope. Scale bars, 5 μm. *n* = 3 per group. The percentage of different sized mitochondria were allocated into three groups: small < 0.75 μm; medium 0.75∼1.25 μm; large > 1.25 μm. GLP-1RA, glucagon-like peptide-1 receptor agonist.

Next, 3 days after the four groups of treatments, we used the fluorescent immunostaining of prohibitin (a kind of mitochondrial scaffold protein) to observe the mitochondrial morphology in the substantia nigra (Figure 6). The morphology of mitochondria was allocated into small, medium, and large groups according to organelle size (small < 0.75 μm; medium 0.75∼1.25 μm; large > 1.25 μm) (Lin et al., 2018; Figures 6A,B). MPTP exposure triggered elongated, condensed, and hyperfused mitochondria, with large mitochondria accounting for 28.34% which indicates that acute MPTP exposure increased a tendency toward the formation of enlarged mitochondria. While in the GLP-1RA group, mitochondria were smaller and more evenly distributed with smaller mitochondria accounting for up to 56.86% while large mitochondria only took up 3.92%. In the MPTP + GLP-1RA group, the mitochondria were smaller and more uniform in size than in the MPTP group. Large mitochondria accounted for 10.53% and small mitochondria accounted for 50%. These data, in combination with the above results of proteins involved in mitochondrial fusion and fission, indicate that MPTP induces mitochondrial enlargement, and the addition of the GLP-1RA normalizes this enlargement. Similar findings have demonstrated that mega-mitochondria are formed under various toxins exposure or unfavorable environments consistent with increasing ROS as an adaptive process (Wakabayashi, 2002). If the cells are additionally exposed to excess amounts of free radicals, these mega-mitochondria will swell, reduce the membrane potential, release cytochrome c, activate caspase, and induce cell death (Seo et al., 2019).

### The GLP-1RA Decreases α-Syn Aggregation and Oxidative Stress Caused by MPTP Exposure in the Substantia Nigra

**FIGURE 7:**
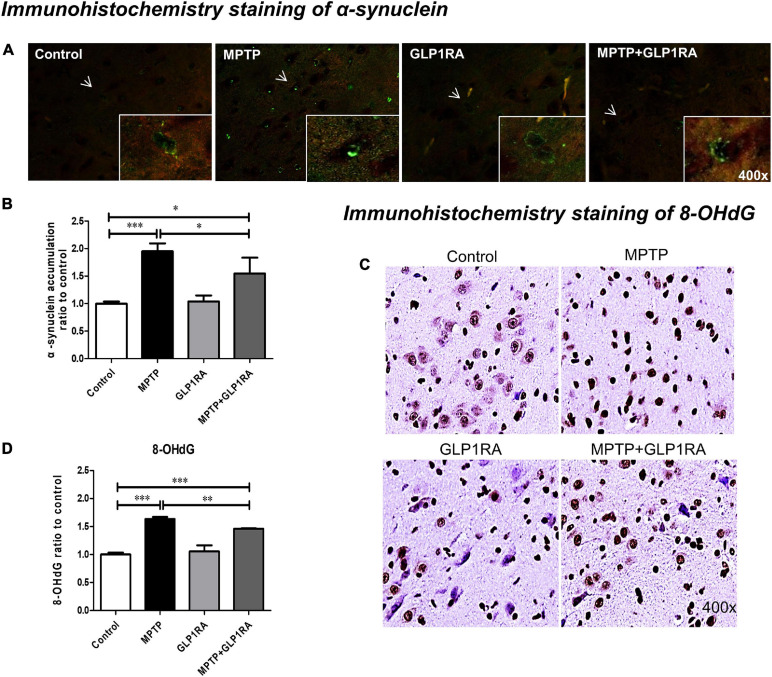
The GLP-1RA (liraglutide) decreases α-synuclein aggregation in substantia nigra and relieves oxidative stress caused by MPTP. IHC staining of α-synuclein (α-syn), the PD hallmark pathological protein, and 8-hydroxy-2-deoxyguanosine (8-OHDG), an oxidative DNA damage marker, in the substantia nigra were performed for the evaluation of pathological α-syn accumulation and oxidative stress levels. Mice were treated according to the four groups, control/MPTP/GLP-1RA/MPTP + GLP-1RA, and sacrificed on the 3rd day. **(A,B)** α-syn-IHC stained substantia nigra sections of the four groups (left to right: the control, MPTP, GLP-1RA, and MPTP + GLP-1RA groups) were viewed via 400x fluorescent microscopy (green) and presented as α-syn staining intensity to percentage of the control, quantified using ImageJ software. Note that representative cells with α-syn accumulation are denoted by arrows. *n* = 3 per group. **(C,D)** 8-OHdG-IHC-stained substantia nigra sections of the four groups (left to right: the control, MPTP, GLP-1RA, and MPTP + GLP-1RA groups) were viewed via 400x light microscopy and presented as 8-OHdG staining intensity to percentage of the control, quantified using ImageJ software. *n* = 3 per group. The values represent mean ± SD (one-way ANOVA, *P < 0.05, **P < 0.01, ***P < 0.001). GLP-1RA, glucagon-like peptide-1 receptor agonist.

Noting the change in mice behavior, cell death, mitochondrial dynamics, and morphology, we probed for further clues of the GLP-1RA effect on MPTP PD models by observing the levels of PD hallmark protein, α-syn, and the correlation with oxidative DNA damage marker, 8-hydroxy-2-deoxyguanosine (8-OHdG) (Figure 7). Immunohistochemistry using an antibody to phosphorylate α-syn, revealed that the exposure of MPTP significantly increased α-syn aggregation by 1.95-fold while the addition of the GLP-1RA to MPTP PD mice decreased the aggregation by 1.55-fold (Figures 7A,B). This is consistent with Vila et al.’s (2000) data that showed that MPTP induced α-syn accumulation in the substantia nigra of mice. Since α-syn aggregation has been shown to be related to oxidative stress, we further quantified 8-OHdG, a predominant form of a free radical-induced oxidative DNA damage marker. The MPTP-exposed group significantly increased 8-OHdG staining by 1.63-fold compared to control and the MPTP + GLP-1RA group decreased this by 1.46-fold (Figures 7C,D). This supports that the GLP-1RA decreased MPTP-induced α-syn aggregation and reduced oxidative stress.

## Discussion

PD, the second most prevalent neurodegenerative disease, has a progressive clinical course, and no neuroprotective strategy is available to mitigate neuronal degeneration. Epidemiological evidence reveals that T2DM patients are more predisposed to acquiring PD (De Pablo-Fernandez et al., 2018). Repurposing of drugs has gained the interest of researchers recently and the neuroprotective effect of the T2DM drugs GLP-1RA on PD has been demonstrated across a range of experimental models of PD (Athauda and Foltynie, 2016). In the current study, we demonstrated that MPTP-caused dopaminergic neurotoxicity can be partially reversed by the GLP-1RA liraglutide in an acute MPTP-induced PD mouse model. Core findings in the present investigation highlight (i) the GLP-1RA significantly alleviated motor impairment in coordination, balance, and exploration induced by MPTP which was reciprocal to the rescue of dopaminergic neurons documented by TH staining over the striatum and substantia nigra; (ii) apoptosis was reduced by the GLP-1RA indicated by the TUNEL stain and cleaved caspase-3 stain; (iii) the GLP-1RA rebalanced disrupted mitochondrial morphology caused by MPTP noted by the reverse effect of fusion and fission protein levels and the immunofluorescent mitochondrial morphology; (iv) perturbed autophagy flux caused by MPTP in dopaminergic neurons was partially reversed by the GLP-1RA; and (v) MPTP enhanced dopaminergic neuron α-syn aggregation which can be reduced with GLP-1RA treatment in correlation with GLP-1RA mitigation of oxidative stress.

The mitochondrial complex I inhibitor MPTP has long been known to reproduce loss of dopaminergic neurons in the substantia nigra with corresponding motor deficits resembling those of PD in primate or rodent PD models (Porras et al., 2012). This neurotoxin MPTP can be converted through monoamine oxidase B (MAO-B) to its toxic-oxidized product MPP^+^ and transported into dopaminergic nerve terminals by the dopaminergic transporter causing the relative vulnerability of dopaminergic neurons to MPTP (Glover et al., 1986). Our neurobehavior data showed that the GLP-1RA liraglutide effectively prolonged the balance time of mice on a RotaRod, increased the times of rearing, and increased total traveling distance and mice exploration in the open field test. Restoration of motor function deficits by the GLP-1RA in behavioral impairment caused by MPTP has been documented similarly (Zhang et al., 2019). The improvements in coordination, balance, and posture in liraglutide-treated animals observed in this experiment may suggest that the GLP-1RA provides protection along motor pathways (Sedelis et al., 2001). Since TH is the rate-limiting enzyme in dopamine synthesis and TH staining is an established marker for dopaminergic neurons (Su et al., 2018), we next observed TH-positive cells in the striatum and substantia nigra to examine if the GLP-1RA could provide nigrostriatal protection from MPTP. In both areas of the brain, the GLP-1RA partially recovered from the dopaminergic neuronal death caused by acute MPTP administration and corresponds to the improved neurobehavior data. This outcome was also described by Liu et al. (2015) and in relation to reduced pro-inflammatory responses. After MPTP convergence to MPP^+^, MPP^+^ is selectively taken up by mitochondria and provokes dopaminergic neurodegeneration through specific inhibition of respiratory chain complex I and consequential induction of mitochondrial-dependent apoptotic cascades (Richardson et al., 2007). To test this hypothesis, we examined the amelioration of dopaminergic neuron death by the GLP-1RA to mitochondria-dependent apoptosis using immunohistochemistry (IHC) stains of apoptosis markers, cleaved caspase 3 and the TUNEL stain (Kühn et al., 2003) in the substantia nigra, the primary site of PD pathology, in experimental mice (Zhang et al., 2020). Our data demonstrated that both cleaved caspase 3 and the TUNEL stain were significantly reduced after GLP-1RA administration to the MPTP PD model which supports the fact that mitochondria-dependent apoptosis is involved in the process of neuroprotection provided by the GLP-1RA.

As highly dynamic organelles, mitochondria constantly undergo coordinated cycles of fusion and fission known as mitochondrial dynamics. These rapid morphological adaptations maintain the integrity of mitochondrial DNA and the balance of oxidative respiration, mitochondrial biosynthesis, and intracellular calcium signaling pathways and also apoptosis (Tilokani et al., 2018; Lin et al., 2020). There are limited studies on the mitochondrial dynamic protein relationship with MPTP; however, MPTP-induced PD models are generally shown to promote mitochondrial fission by increasing fission protein Drp1 and decreasing fusion protein OPA1 leading to a fragmented phenotype (Park et al., 2019; Valdinocci et al., 2019). Exploration focused on the effect of the GLP-1RA in mitochondrial morphology alternation is even more scarce. Here we examined the effect of the GLP-1RA on major regulatory proteins involved in mitochondrial dynamics and mitochondrial morphology in the substantia nigra of an MPTP PD model. Our data showed that MPTP decreased OPA1, the key player regulating MIM fusion, which was re-elevated with the addition of the GLP-1RA. However, to our surprise, our data for the levels of MOM fusion proteins, MFN1 and 2, were increased in MPTP-treated mice while the addition of the GLP-1RA lowered MFN1 and 2 levels. Reciprocally, the active form of fission protein p-Drp1 was decreased in the MPTP-treated group while the GLP-1RA elevated this protein level. These findings differ from general phenomenon as demonstrated by Manqing et al. (2019) in an MPTP rodent model where they only studied the mitochondrial morphology regulators in the striatum of mice which is not the major pathologic degeneration locus in PD pathology. These data may be a result of the different timing of observations and different dosing and administration duration time of MPTP in variable tissue locations. Our data suggested that MPTP and the GLP-1RA work in counter mechanisms and that the morphological mitochondrial proteins were dynamically changed. These results imply that mitochondrial dynamic balance is essential for neuronal survival under stress induced by MPP^+^, and restoration of mitochondrial morphology alteration through the GLP-1RA may provide further protection. Mitochondrial dynamics include the balance of mitochondrial morphology modification and the regulation of mitochondrial quality control mechanism through selective clearance of damaged mitochondria via autophagy, termed mitophagy (Xie et al., 2020). Recently, genetic findings implicate that the autophagy-lysosome system, the terminal process of mitophagy, is critically involved in PD pathogenesis (Okarmus et al., 2020). In the process of autophagy, the conversion of LC3-I (free form) to LC3-II (phosphatidylethanolamine-conjugated form) represents a key step in autophagosome formation in mammals (Chen et al., 2010). p62/SQSTM1 is a selective autophagy adaptor that shuttles ubiquitinated proteins to autophagosomes or proteasomes by recognizing LC3-II (He et al., 2018; Jung and Oh, 2019). In our previous study, we have reported impaired autophagy flux in a similar rotenone complex I inhibition cellular PD model. This observation was documented by elevation of both LC3-II and p62 expression in rotenone-triggered neurotoxicity which can be counterbalanced with the addition of the anti-oxidant resveratrol which significantly reduced the accumulated p62 (Lin et al., 2018). Here, we demonstrated that MPTP elevated p62 accumulation but decreased the LC3-II/LC3-I ratio suggesting an impeded autophagy flux without induction of autophagosome formation which may lead to further accumulation of damaged organelles. The addition of the GLP-1RA to MPTP-treated mice lowered p62 levels and elevated the LC3-II/LC3-I ratio which suggests that augmentation of autophagy flux provides neuroprotective effect in this model. This observation is consistent with Hu et al.’s (2017) finding that MPTP blocked autophagy flux in zebrafish. The protective effect of the GLP-1RA through the elevation of autophagy flux in an acute MPTP exposure rodent model is limited, although in a chronic MPTP model the autophagy flux was not altered significantly post GLP-1RA treatment (Zhang et al., 2019), while this has been reported in pancreatic β-cells by Zummo et al. (2017). We further observed mitochondrial morphology under a fluorescent microscope since morphology-related protein levels can only provide information in a time cross-sectional manner. Not surprisingly, we found morphological manifestations corresponding to the protein levels. Under acute 4-dose MPTP exposure, instead of noting fragmented mitochondrial morphology as according to the majority of documentation, we noted hyperfused, condensed, and elongated mitochondria on the third day. The MPTP + GL-1RA group, on the other hand, normalized morphological alteration caused by MPTP exposure. Mega-mitochondria, a phenomenon of significantly enlarged mitochondria beyond mere elongation, have been observed in pathological conditions causing the imbalance of mitochondrial dynamics and elevated oxidative stress (Zhou et al., 2020). A recent report suggests mega-mitochondria formation to be an adaptive response to an unfavorable environment, where mitochondria go through extended fusion in an attempt to reduce ATP generation, consume less oxygen, and ultimately decrease ROS production (Wakabayashi, 2002). If mitochondria succeed to suppress intracellular ROS levels, the mitochondrial network can return to normal both structurally and functionally. Should excessive stress continue and mitochondria become beyond repair, mega-mitochondria may induce apoptosis subsequently (Jiang et al., 2019). Interestingly, Ziviani et al. (2010) showed that the inactivation of PINK1/parkin, familial PD genes, suppresses the ubiquitination of MFN causing impeded parkin/PINK1-mediated mitophagy, leading to the formation of mega-mitochondria. Palma et al. (2019) also demonstrated that the inhibition of Drp1 drives mega-mitochondria formation as an adaptive response in alcohol-induced hepatotoxicity in hepatoma cells. Therefore, we further investigated if the oxidative stress marker 8-OHdG and the most characteristic PD pathogenic protein α-syn were elevated in the MPTP group in substantia nigra tissue and whether this can be recovered through the usage of the GLP-1RA. Mitochondria are the primary source of ROS and the proximity of these highly reactive free radicals may further cause damage to mitochondrial macromolecules including proteins and mtDNA (Subramaniam and Chesselet, 2013). Oxidative stress is also shown to contribute to the cascade leading to nigrostriatal dopaminergic neurodegeneration in PD (Desai et al., 1996; Ekstrand et al., 2007; Dias et al., 2013). In our data, the elevation of oxidative stress in the MPTP group was evident by 8-OHdG staining, a predominant form of a free radical-induced oxidative lesion in DNA (Chung et al., 2017), and the addition of the GLP-1RA significantly decreased 8-OHdG staining. Evidence has demonstrated that α-syn aggregation can be exacerbated with compromising free radical scavenging capacity which suggests elevated levels of oxidative stress may modulate α-syn aggregation and the progression of PD (Scudamore and Ciossek, 2018). Studies looking at the effect of the GLP-1RA on α-syn aggregation in PD models are limited, though in one chronic low dose MPTP mice PD model (20 mg/kg/day), α-syn aggregation was reduced after 30 days of GLP-1RA administration (Zhang et al., 2019). Here we showed that α-syn aggregation correlates to oxidative stress data and while MPTP increased α-syn accumulation, the GLP-1RA reduced the level of this toxic protein in an acute MPTP PD model (80 mg/kg in 4 doses administrated 2 h apart).

**FIGURE 8:**
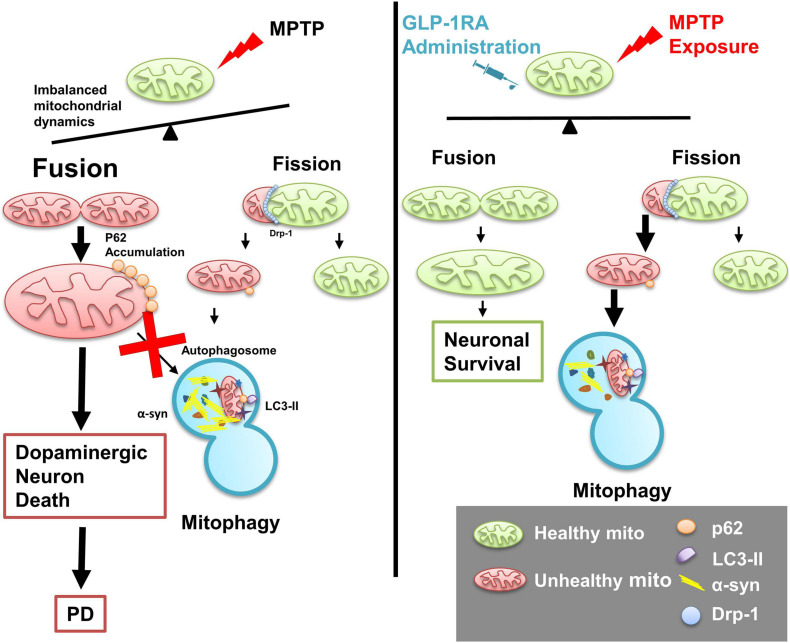
Schematic diagram of the role of the GLP-1RA (liraglutide) in mediating MPTP-induced imbalanced mitochondrial fusion and fission and rescuing neurotoxin-induced neuronal death. In a PD mouse model, the mitochondrial complex I inhibitor MPTP causes loss of dopaminergic neurons and imbalance of regulators involved in mitochondrial morphology dynamics. Enlarged mitochondria (mega-mitochondria) are formed as an adaptive process under stress and apoptosis is induced once the stress is overwhelming, and reduced autophagosomal formation is associated with enhanced α-syn accumulation and elevated oxidative stress. The T2DM drug GLP-1RA, liraglutide, rebalanced mitochondrial dynamics, enhanced autophagy flux, and provided a neuroprotective effect. Mito, mitochondria; p62, p62/Sequestosome 1 (SQSTM1); LC3-II, microtubule-associated protein 1A/1B-light chain 3; α-syn, α-synuclein; Drp1, dynamin-related protein 1.

In summary, our study provides comprehensive coverage on the neuroprotective effect of the GLP-1RA, liraglutide, in an acute MPTP PD mouse model. Acute MPTP exposure causes mitochondrial morphology alteration and the mega-mitochondria formation associated with impaired autophagy flux leading to dopaminergic neuronal loss and motor deficit in experimental mice. Liraglutide partially improves neurobehavior performance and decreases dopaminergic cell death through reducing apoptosis, rebalancing mitochondrial dynamics, regulating mitophagy with enhancement of autophagy flux, mitigating α-syn accumulation, and reducing oxidative stress in the substantia nigra of the PD mouse model (Figure 8). Our investigative results shed new light on repurposing a widely used clinical drug GLP-1RA on PD patients through balancing mitochondrial morphology and enhancing autophagy flux.

## Data Availability Statement

The raw data supporting the conclusions of this article will be made available by the authors, without undue reservation.

## Ethics Statement

The animal study was reviewed and approved by the Institutional Animal Care and Use Committee (IACUC).

## Author Contributions

T-KL contributed to concept generation, performing the experiments, data collection, data interpretation, drafting and editing of the manuscript, and graphic drawing. K-JL contributed to concept generation, data interpretation, drafting of the manuscript, and graphic drawing. H-YL and P-CT contributed to performing the experiments, data interpretation, and drafting of the manuscript. K-LL contributed to concept generation, data interpretation, and drafting of the manuscript. M-YL, P-WW, T-JW, and F-SW contributed to concept generation and data interpretation. C-WL and J-HC contributed to concept generation, data interpretation, editing of the manuscript, and approval of the article. All authors contributed to the article and approved the submitted version.

## Conflict of Interest

The authors declare that the research was conducted in the absence of any commercial or financial relationships that could be construed as a potential conflict of interest.
